# Building a Network of Health Professionals for Breast and Cervical Cancer Control in the Andean Region

**DOI:** 10.1200/JGO.2016.008714

**Published:** 2017-06-12

**Authors:** Diana Mendoza-Cervantes, Isabel Otero, Jo Anne Zujewski, Jorge Ferrandiz Salazar, Gabriela López Córdova, Cathy Muha, Lisa Stevens

**Affiliations:** **Diana Mendoza-Cervantes**, **Isabel Otero**, **Jo Anne Zujewski**, **Cathy Muha**, and **Lisa Stevens**, National Institutes of Health, Rockville, MD; and **Jorge Ferrandiz Salazar** and **Gabriela López Córdova**, Ministerio de Salud de Perú, Jesús María, Lima, Peru

## Abstract

**Purpose:**

Cancer mortality is approximately twice as high in Latin American countries than in more developed countries. In particular, the countries of the high Andean region of Latin America carry a double burden of breast and cervical cancers. In these countries, there are disproportionately higher mortality to incidence ratios compared with other regions in Latin America. The US National Cancer Institute’s Center for Global Health, the Pan American Health Organization, and the Ministry of Health in Peru collaborated to design and execute an education and advocacy workshop in Lima, Peru. The workshop was convened to discuss regional challenges and practices, as well as to support the implementation of Plan Esperanza, Peru’s national cancer control plan.

**Methods:**

Workshop participants included local and international experts to present the state of the science, health practitioners, and advocacy groups to discuss unique barriers that women in the region experience.

**Results:**

Inequalities in access to and distribution of medical expertise, lack of continuity of cancer control plans, and the need for sustained public buy-in emerged as obstacles.

**Conclusion:**

The workshop provided a forum to discuss key issues regarding breast and cervical cancer control among health professionals and advocates in Peru and the region. This article outlines the resulting recommendations.

## INTRODUCTION

Breast and cervical cancers are the leading cause of cancer deaths in women worldwide.^1^ The growing cancer burden is driven in part by social determinants of health such as sex, ethnicity, and socioeconomic status, which are influenced by rising socioeconomic disparities in the western hemisphere.^2,3^ The 2015 Lancet Oncology Commission, which presented the progress of cancer in Latin America, identified disparities in cancer control as a central area of concern. The commission recommends, among other things, increasing oncology workforce training, extending initiatives to train health care personnel in remote areas, addressing disparities in the concentration of cancer services and expertise in urban areas, and customizing strategies for cancer screening to fit local resources.^2^ Health education and advocacy have repeatedly been called for to tackle the barriers that prevent women from seeking health care in populations with rising socioeconomic inequalities, such as in the high Andean region of Latin America.^4,5^ One recommendation for addressing the needs of fragmented populations calls for increased efforts to “bridge language, social, and cultural gaps between patients and oncology providers.”^6(p403)^ High-level recommendations for national cancer control plans (NCCPs) aim to bridge such gaps in equitable services.

An NCCP is a public health program that requires multisectorial strategies. According to the WHO, an NCCP aims to reduce cancer incidence and mortality while improving the quality of life of patients “through the systematic and equitable implementation of evidence-based strategies for prevention, early detection, diagnosis, treatment, and palliation.”^7(pix)^ Cancer control plans specifically address inequalities in access to health care, knowledge, and technical deficits; they also ensure planning of continuity of cancer control programs. These factors are key to reducing mortality and morbidity as the result of breast and cervical cancers in the region.

Efforts to implement effective breast and cervical cancer prevention and control programs have not been successful in all countries. However, Peru stands out as a leader in the region for its achievement in executing its NCCP: Plan Nacional para la atención Integral del cáncer y mejoramiento del acceso a los servicios oncológicos en el Perú—Plan Esperanza. Peru’s process began with a multisectorial coalition, “Peru Against Cancer,” which was formed in 2005 with the aid of international organizations. The coalition led to development of an NCCP, with the aim to eradicate advanced cancers, implement health education, and provide access to services for cancer control by 2016. Reducing the rates of breast and cervical cancers is a top priority in Peru’s cancer control plan, which also includes a plan for universal insurance coverage.^8^ These policies led to the 2012 launch of Plan Esperanza, with the main objective to improve access to cancer care services. The launch was accompanied by a massive investment by the Peruvian government of $290 million to cover the cost of treatment of patients with low income.^8,9^ The emphasis of the cancer plan on decentralization has shifted decision making to the local community, highlighting the critical role of advocacy and education at the local level. Recommendations from the recent 2015 Lancet Oncology Commission, the WHO, and the Breast Health Global Initiative echo the move toward decentralization as they call for clinical downstaging as a more cost-effective solution. This strategy consists of raising the awareness of early signs and symptoms in the public, educating first-line health professionals, and improving referral procedures to enable prompt, adequate diagnosis and treatment of cancer at early stages.^6^

When describing the priorities for the health system in the new administration, Peru’s Minister of Health, Patricia García, stresses the need to focus specifically on primary care to combat chronic disease and to improve coordination between national and regional service centers.^10^ The Peruvian health care system is organized by three levels of care. At the first level, health providers include general practitioners, nurses, obstetricians, and technicians; there is a subdivision within the first level depending on the complexity of the health facility. Specialists such as those working in pediatrics, gynecology, internal medicine, and pathology comprise the second level. Finally, practitioners who specialize in GI medicine, oncology, neurology, and other similarly complex areas of care comprise the third level. The Peruvian Ministry of Health and the Peruvian National Cancer Institute normally train providers with an emphasis on prevention. Regional cancer coordinators usually develop health management and cancer prevention skills. Nonetheless, some of them practice without formal background and training in these areas.

Since Peru began to implement Plan Esperanza, it has successfully integrated the health sector at local, national, and regional levels, with multidisciplinary teams including the Peruvian government and civil society.^11,12^ Importantly, although the plan does not specifically address training, the Ministry of Health trained health care personnel in breast and cervical cancer prevention using the Papanicolaou test and visual inspection with acetic acid. Additionally, innovations in training were recently included in the cervical cancer prevention and care guidelines, which introduce human papilloma virus primary screening, including a virtual training course for health care providers at the primary level. Although there is a growing base of advocacy groups that work on defending patients’ rights to Peruvian health institutions, the need to further integrate advocates in the development and implementation of cancer control efforts, such as by obtaining their input to validate guidelines, remains.

The Instituto Nacional de Enfermedades Neoplásicas (INEN) often works with the Peruvian National Institute of Health and the Ministry of Health of Peru. In recent years, they have also connected with scientific networks and international health organizations to strengthen the implementation of Plan Esperanza.^12^ In this joint effort, the US National Cancer Institute, the Ministry of Health, and the Pan American Health Organization (PAHO) held a multisectorial workshop on breast and cervical cancer education and advocacy, which convened scientific experts from the United States and Latin America. The priorities of the workshop were disseminating scientific evidence for best practices in breast and cervical cancer control, supporting the advancement of Peru’s efforts for equitable cancer control, and building a regional network of health professionals and advocates for breast and cervical cancer control in the high Andean region.

The workshop also provided the National Cancer Institute’s collaborators with the opportunity to use the Knowledge Summaries for Comprehensive Breast Cancer Control (KSBCs) as educational materials. The KSBCs are directed toward policy makers on the basis of the framework of the Breast Health Global Initiative resource-stratified pathways.^13^ The toolkit addresses foundational issues in comprehensive breast cancer care across the cancer continuum, recognizing that health systems vary significantly around the globe. In addition, as a care pathway, the KSBCs serve as a communication tool, as well as provide evidence-based research on the integration of services and resource use and prioritization. The entire set is available in English, and four knowledge summaries are provided in Spanish; these can be accessed online.^14^

This article discusses how the three priorities were addressed during the workshop and how the open forum discussions resulted in recommendations for the region. First, when disseminating scientific evidence, there is a need to address the unequal concentration of evidence-based programs, medical technology, and expertise in developed urban areas compared with rural areas. Second, in leveraging Peru’s success with Plan Esperanza, there is a need to emphasize the importance of working across sectors (to include the Ministry of Finance) to ensure the continuity of cancer control programs through changes in leadership and health investment priorities. Finally, by providing guidance and facilitation for advocates and survivors from the region, the workshop revealed various approaches to sustaining momentum for public buy-in.

## METHODS

The US National Cancer Institute, PAHO, and the Ministry of Health of Peru designed and implemented the workshop on breast and cervical cancer education and advocacy to show how the work in Peru could serve as a model for the region. Following recommendations from PAHO, the Ministry of Health of Peru, and INEN, national and regional experts in breast and cervical cancers were identified for each session’s presentations. Workshop participants included a comprehensive range of stakeholders, including policy makers, program managers, researchers, health care providers, advocates, and cancer survivors (Table 1). Advocacy and civil society organizations from Bolivia, Colombia, Ecuador, Venezuela, and Mexico were also invited and participated in the workshop. Experts from local and international health institutions presented the didactic sessions, and advocates and patient survivors presented their work and experiences through a panel; however, all participants contributed to the general discussions.

**Table 1:**
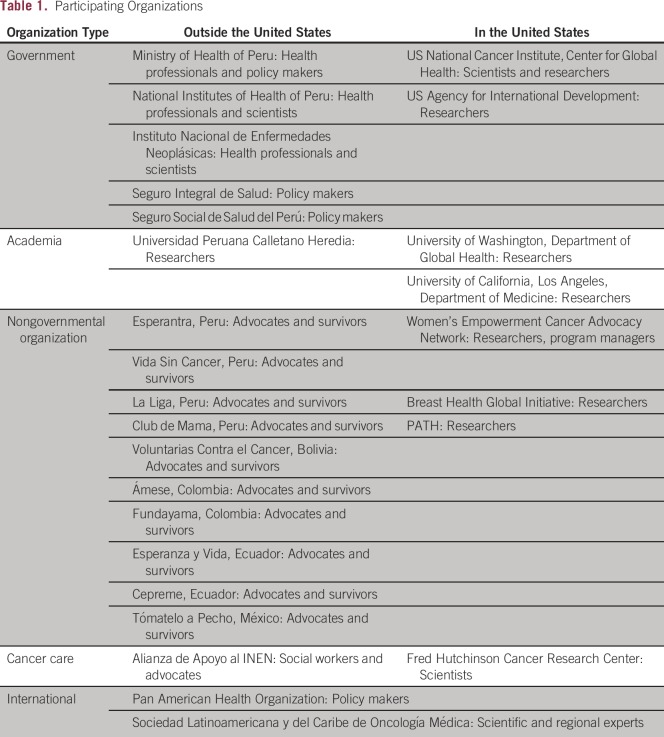
Participating Organizations

In addition, the Ministry of Health of Peru nominated public health practitioners and regional cancer care coordinators in Peru, which included physicians, nurses, and other health care workers. A total of 63 participants from 26 regional districts in Peru were trained (Table 2). Within this cohort were professionals who perform social service work by supporting health centers that are designated as training centers, provide gynecologic cancer prevention services, and train health professionals at other establishments. Many health care providers also serve important functions in public health because they are responsible for the coordination of their regional and local cancer control strategies (Table 3). In addition, the workshop training counted toward the accreditation of the cancer care coordinators nominated by the Ministry of Health. For this effort, the National Cancer Institute provided technical assistance and, with representatives from the PAHO, the Ministry of Health, and INEN, developed a curriculum that was tailored to the needs of local health care providers.

**Table 2:**
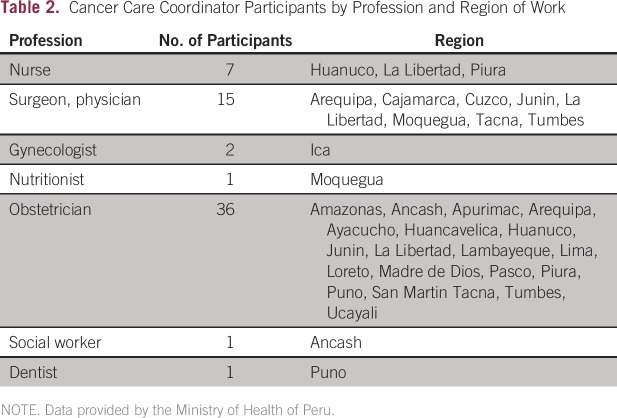
Cancer Care Coordinator Participants by Profession and Region of Work

**Table 3:**
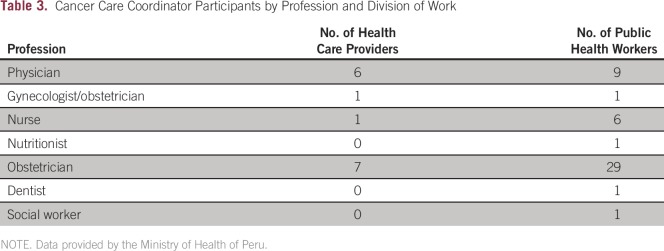
Cancer Care Coordinator Participants by Profession and Division of Work

The workshop sessions included presentations of scientific evidence, panels to share international experiences, active participation in lectures on breast and cervical cancer control across the cancer continuum, and distribution of KSBCs that were piloted for dissemination in low- and middle-income countries. While the National Cancer Institute contributed technical assistance, the Fred Hutchinson Research Center led data collection for evaluation of the KSBCs. Other collaborators included the University of Washington, the Universidad Peruana Cayetano Heredia in Peru, and the Women’s Empowerment Cancer Advocacy program for stigma and advocacy research.

The distinct sessions were structured as follows. Day 1 included the state of breast and cervical cancer control in the high Andean region and a cervical cancer training session that included the experience of various groups. Day 2 focused on breast cancer training and the pre- and postevaluation of the KSBCs. Day 3 focused on stigma, mobilizing advocacy groups and civil society, and included a panel in which cancer survivors presented their experiences. An open forum discussion, which was facilitated by representatives from PAHO, the Ministry of Health, and the National Cancer Institute, concluded each of these sessions; written notes were recorded from active participation in the lectures as well as the general discussion. Finally, groups of practitioners, advocates, and survivors participated in focus groups and individual interviews about various topics related to women’s cancer control and advocacy, which were led by the University of Washington. The results of this analysis will be published in a separate article. At the end of the workshop, the points of discussion were disseminated to the public via local news as well as the Web sites of the PAHO and the National Cancer Institute. At the end of the meeting, all participants received flash drives with the materials for the workshop, including a participant list, KSBCs, and an advocacy toolkit. Additionally, networking lunches were designed to encourage participants from diverse backgrounds to meet and discuss their specific areas of interest.

## RESULTS

Most of the attendees participated in all sessions and were encouraged to continue discussions during the networking breaks. On the basis of conversations with the Ministry of Health, some of the cancer coordinators subsequently trained others in their regional health centers after the conference. The advocates and cancer survivors initiated a messaging group through an established application to share their experiences during campaigns and to keep in contact after the meeting. In addition, advocates from Bolivia, which does not have an NCCP, were motivated to develop and share with the National Cancer Institute a draft proposal for legislation on an integrated approach to cancer control in Bolivia. After the workshop, advocates reported that they collaborated and arranged visits to different organizations to share best practices. Groups such as the Voluntarias Contra el Cancer from Bolivia intended to use the training to advocate for national policy changes. Didactic presentations encouraged active participation from all attendees. Professionals invested in different phases of cancer care actively contributed their valuable perspectives during each discussion session.

### Recommendations

The open forum discussions included valuable contributions from stakeholders and experts across the continuum of care. The following recommendations for improving women’s cancer care and control in Peru and the region resulted from this process.

#### Multidisciplinary learning and communication.

A lesson learned was the importance of collaboration among distinct health professionals to ensure improved outcomes across the continuum. Convening scientific experts, policy makers, care providers from remote and central areas, patient advocates, as well as cancer survivors to share their unique perspectives, revealed that the role of multidisciplinary communication in this complex work is fundamental. Didactic sessions drew from international guidelines, scientific evidence, and the practices of organizations such as PATH, which have experience conducting pilot studies in the region for various technologies and care pathways. This design allowed policy makers and health care providers to learn from the experiences of patient survivors and advocates in overcoming barriers to patient navigation, preventing loss to follow-up, dispelling stigmas, and catalyzing key messages for health promotion in remote areas.^15^ Although most health care providers were familiar with some of the barriers to care, a comment from a health care worker specifically stressed a different problem. Paraphrased, the worker said that health professionals who lack communicative procedures to overcome differences in socioeconomic and educational status can exacerbate the barriers to care. Input from health care providers, nongovernmental organizations, and policy makers is necessary to address the experiences of patients and survivors. These experiences are key in addressing the disparities in cancer care available to women across the region and should be included in NCCPs as part of a multidisciplinary learning strategy.

#### Integrating advocacy and civil society groups to improve access and to prevent loss to follow-up.

A shared challenge, which was identified in focus groups and interviews with advocates and cancer survivors from the high Andean region, was the need to better communicate the value of the cancer advocate in the health care system. A direct Spanish translation of the word “advocate” is not in common use. However, beginning to define this role is a necessary step toward empowering women, as well as communities of survivors, to share their narratives, dispel stigmas, and freely advocate for necessary policy changes that would increase access to health services for women of all backgrounds. During the sessions, a community Peruvian health care provider confirmed the belief that “it is fear, definitely they think that if they have cancer or if we are going to diagnose cancer they are going to die, there is no survival.” Advocacy and civil society groups work to dispel similar stigmas and should be integrated into the process of designing and implementing cancer control programs.

In addition, the roles of advocacy and civil society groups should be recognized in the process of translating the rapidly evolving scientific evidence for local implementation and use. Empowering advocates, in particular, helps engage the public in dispelling harmful stigmas. Additionally, advocacy is integral to building public trust in ongoing efforts and has been articulated as a critical consideration in preventing loss to follow-up of women in areas with long time lapses between screening, diagnosis, and treatment.^16^ Loss to follow-up may also result from misconceptions, as well as from seemingly practical reasons given by women with various family and work obligations who are likely to disregard their own health needs.^16,17^ Peru has implemented solutions such as mobile campaigns to distant communities, limiting visits (by offering screening and immediate treatment during the same visit), and efforts to improve the referral system.^18^ The integration of advocates and civil society, which are gaining momentum in the work with medical institutions, in Peru specifically, seeks to improve such approaches by tackling myths and misconceptions. This is a process that begins locally within a community and is extended with national and regional advocacy networks. Tackling breast and cervical cancer control in low- and middle-income countries is an issue at the intersection of human rights and health policy, and it calls for cancer control policies to seriously consider patients’ realities.^19^ In addition to including evidence-based approaches to health promotion, the development of such policies should be informed by affected individuals. The work advanced by Plan Esperanza shares these priorities for Peru and hopefully influences similar changes in the region.^20^

#### Committing to equitable breast and cervical cancer control.

Recognizing the link between prevention and control of breast and cervical cancer, women’s empowerment, and subsequent increased economic participation in society is key to sustainably and equitably reducing the cancer burden. These factors come into focus as a majority of women who suffer the effects of breast and cervical cancers are at an age when they lead productive lives, often function as primary caregivers, have careers, or run their households. In this context, a cancer diagnosis is destabilizing. A survivor who considered selling her house described the uncertainty she felt for her family: “I was worried about my children [because] they had not yet established themselves…I suffered financially and physically and emotionally.” A second patient survivor emphasized the pressure of carrying the burdens of the disease: “I didn’t even have the courage to tell [my family] I had cancer.” For many women, their well-being is integral to their family’s welfare, as well as to social development of the community.

Since 2013, Plan Esperanza has made significant advances in mobilizing multiple sectors that are heavily investing in cancer, ensuring health coverage for socioeconomically disadvantaged individuals who are diagnosed with cancer, and empowering authorities and health workers with enhanced knowledge in cancer prevention.^11,21^ The workshop played a role in extending these effects and helped develop a regional network for professionals committed to advancing prevention and control of breast and cervical cancers in women.

### Limitations

A main limitation includes the invitation-only participant list, which may have prevented perceptions and barriers that are present in other demographics from being expressed. For example, greater representation from a more diverse group of patients, and from policy makers who work outside of the health sector but who may be able to recognize and influence specific economic and political policies that affect health care systems, would have been valuable to the discussion.

To summarize, advocates are key drivers of health communications. They change perceptions that have the potential to influence social norms, prompt action, and demonstrate necessary behavioral changes for society. Advocates should thus be empowered as primary influencers for policy making and be considered especially critical in preventing loss to follow-up across the continuum of breast and cervical cancer care. Their work, however, must be construed as part of a larger multisectorial effort to integrate scientific evidence in the development and implementation of NCCPs.^17^

Many barriers to proper breast and cervical cancer prevention, diagnosis, and treatment are rooted in disparities on the basis of ethnicity, sex, and socioeconomic status, among other factors. Ensuring equitable breast and cervical cancer prevention and care in NCCPs, in addition to building a network of health professionals and advocates, presents a strategic opportunity to reduce the overall cancer burden as well as improve the quality of life of many women. These factors enhance the empowerment of women and their contributions to societal development, while averting death and suffering.

In conclusion, the work of breast and cervical cancer prevention and control comprises both economic and human rights imperatives.^22,23^ It requires collaboration among health professionals and advocates at every stage of the continuum of care. The workshop leveraged the growing momentum in Peru—a leader in the region as evidenced by the evolution of Plan Esperanza, sustained government support, and public buy-in—to exchange insights within and across health systems and to catalyze the reduction of mortality and morbidity as the result of breast and cervical cancers in the region.
